# Correction: Intravascular flow stimulates PKD2 (polycystin-2) channels in endothelial cells to reduce blood pressure

**DOI:** 10.7554/eLife.60401

**Published:** 2020-06-30

**Authors:** Charles E MacKay, M Dennis Leo, Carlos Fernández-Peña, Raquibul Hasan, Wen Yin, Alejandro Mata-Daboin, Simon Bulley, Jesse Gammons, Salvatore Mancarella, Jonathan H Jaggar

MacKay CE, Leo MD, Fernández-Peña C, Hasan R, Yin W, Mata-Daboin A, Bulley S, Gammons J, Mancarella S, Jaggar JH. 2020. Intravascular flow stimulates PKD2 (polycystin-2) channels in endothelial cells to reduce blood pressure. *eLife* **9**:e56655. doi: 10.7554/eLife.56655.

Published 4, May 2020

It has come to our attention that we made a typographical error in reporting catalog numbers in this article related to the anti-PKD2 antibodies. The mouse monoclonal anti-PKD2 primary antibody from Santa Cruz was originally listed incorrectly as catalog number sc-100415. We in fact used two different catalog numbers for the mouse monoclonal anti-PKD2 primary antibodies from Santa Cruz. The *correct* catalog numbers for these antibodies are sc-28331 and sc-47734.

We also stated that the anti-p-eNOS antibody obtained from Cell Signaling Technology was a rabbit monoclonal, when it was a rabbit polyclonal.

The article has been corrected accordingly. We apologize for the errors.

